# Phylogenetic placement of thermophilic ammonium-tolerant bacteria and their distribution in various composts

**DOI:** 10.5713/ab.22.0216

**Published:** 2022-09-07

**Authors:** Kazutaka Kuroda

**Affiliations:** 1 Division of Livestock Research, Kyushu Okinawa Agricultural Research Center, National Agriculture and Food Research Organization, Koshi 861-1192, Japan

**Keywords:** Adaptability, Ammonia, Ammonium Nitrogen, Composting, Thermophilic Ammonium-tolerant Bacteria

## Abstract

**Objective:**

Previous studies isolated the thermophilic ammonium-tolerant (TAT) bacterium *Bacillus* sp. TAT105 that grew in composting swine manure with the assimilation of ammonium nitrogen and reduced ammonia emissions during composting. Those studies also investigated the potential for applications of TAT105 to composting. It was observed that the concentration of TAT bacteria, phylogenetically close to TAT105, increased during composting. The objectives of this study were to identify the phylogenetic placement of these TAT bacteria and investigate their distribution in various composts.

**Methods:**

The phylogenetic placement of TAT105 was examined based on the sequence of 16S ribosomal RNA gene. The genomic DNA homology between TAT105 and the type strains of bacterial species that were phylogenetically close to TAT105 were examined by DNA–DNA hybridization. Moreover, the tolerances of these strains to NH_4_Cl and NaCl were analyzed using a cultivation method. Concentrations of TAT bacteria in various composts were evaluated using an agar medium specific to TAT bacteria and polymerase chain reaction followed by restriction fragment length polymorphism analysis.

**Results:**

TAT105 was most closely related to *Bacillus thermolactis* and *Bacillus kokeshiiformis*. Many variants of these species have been detected in various environments, including composts. The type strains of these species displayed TAT characteristics that were similar to those of TAT105. Among the composts examined in this study, TAT bacteria were detected at high concentrations (10^5^ to 10^9^ colony forming units per gram of dry matter) in most of the composts made from cattle manure, swine manure, bark, and excess sludge.

**Conclusion:**

TAT bacteria comprised *B. thermolactis*, *B. kokeshiiformis*, and their phylogenetically close relatives. They were considered to be adaptable to composting of some certain materials, and a favorable target for searching for strains with some useful function that could be applied to composting of these materials.

## INTRODUCTION

Composting is a treatment method for various organic wastes to produce compost used as a plant fertilizer or soil conditioner, and it is popularly used as a treatment for animal manure [1,2]. During composting, microorganisms actively decompose and stabilize the organic matter in manure. This process generates large amounts of heat and various metabolites, including water, carbon dioxide, other greenhouse gases, and malodorous compounds [2–4]. Many microorganisms are involved in composting, and they have been studied for various purposes, including searching for microorganisms with useful functions and their applications to composting: reducing odor emission [5–7], preparing plant pathogen-suppressive compost [8,9], and increasing the rate of organic matter decomposition [10].

Ammonia (NH_3_) is a metabolite and a representative malodorous compound generated from the decomposition of organic matter. It is largely emitted during the composting of animal manure [2–4], and causes various environmental problems, as well as complaints against malodor [3,11]. A previous study isolated a thermophilic ammonium-tolerant (TAT) bacterium *Bacillus* sp. TAT105, a thermophile (temperature range 40°C to 65°C), from a compost prepared from livestock manure [12]. This bacterium had a high tolerance to NH_4_Cl (≤1,200 mM), and grew on swine manure assimilating ammonium nitrogen (NH_4_-N). Phylogenetic analysis based on the sequence of 16S ribosomal RNA gene (16S rDNA) showed that TAT105 is closely related to several thermophilic bacilli: *Bacillus thermoamylovorans*, *Bacillus aestuarii*, *Bacillus fumarioli*, and *Bacillus smithii*. In contrast, polymerase chain reaction followed by restriction fragment length polymorphism analysis (PCR-RFLP) of 16S rDNA using two restriction enzymes (HaeIII and HinfI) revealed that PCR-RFLP profiles of HaeIII and HinfI digests of 16S rDNA from TAT105 differed from those of the thermophilic bacilli listed above [13].

The previous studies also examined the application of this bacterium for reducing NH_3_ emissions in composting of swine manure [12–14]. In the composting tests in these studies, two treatments were included: one in which TAT105 was added to swine manure (TAT-added) and another without TAT105 (Control). Reductions in NH_3_ emissions and nitrogen loss from manure during composting were confirmed in TAT-added compared with Control in repeated tests. In these studies, a cultivation method for detecting TAT bacteria on agar medium containing 1 M NH_4_Cl was developed, and using this method, concentrations of TAT bacteria in composted manure were estimated during the composting tests [13,14]. In TAT-added, TAT bacterial concentrations in the manure were controlled to approximately 10^7^ colony forming units per gram of dry matter (CFU/g dry matter [DM]) at the start of composting by the addition of TAT105, which increased to 10^9^ to 10^10^ CFU/g DM by the end of composting. TAT bacteria were also detected in Control without adding TAT105 at low concentrations (<10^5^ CFU/g DM) at the start of composting, and the concentrations increased to 10^8^ to 10^9^ CFU/g DM by the end of the experiment. Some of the colonized TAT bacteria on the agar were isolated and subjected to amplification of 16S rDNA by PCR-RFLP. The PCR-RFLP profiles of HaeIII and HinfI digests of these TAT bacteria were same as those of TAT105 [13]. These results suggested that TAT bacteria that were phylogenetically close to TAT105 indigenously existed in swine manure and had a high adaptability to composting.

The sequence databases are constantly updated, and the genus *Bacillus* and its related genera have been expanded. Revision of the phylogenetic placement of TAT105 should provide more detailed information about the TAT bacteria. In this study, phylogenetic analyses of TAT105 were conducted based on the recent expansion of *Bacillus* spp. to verify the phylogenetic placement of the TAT bacteria. In addition, the distribution of TAT bacteria in composts prepared from various materials was investigated.

## MATERIALS AND METHODS

### Phylogenetic analyses of TAT105 and its close relatives

The 16S rDNA sequences in the International Nucleotide Sequence Database Collaboration (INSDC) with high similarity to that of TAT105 were identified using the BLAST algorithm of the National Center for Biotechnology Information (Bethesda, MD, USA). Phylogenetic trees based on the neighbor-joining method [15] were constructed using Clustal X, a software application for multiple sequence alignment [16]. Restriction sites for HaeIII and HinfI in 16S rDNA were confirmed using the NEB cutter V2.0, an online tool to identify restriction sites in DNA sequence (New England BioLabs. Inc., Ipswich, MA, USA; http://nc2.neb.com/NEBcutter2/).

The type strains of the two phylogenetically closest relatives of TAT105, *Bacillus kokeshiiformis* strain MO-04 (JCM 19325^T^) and *Bacillus thermolactis* strain R-6488^T^ (DSM 23332^T^), were purchased from the Japan Collection of Microorganisms (Tsukuba, Japan) and Deutsche Sammlung von Mikroorganismen und Zellkulturen GmbH (Braunschweig, Germany), respectively.

The homologies of genomic DNA between TAT105 and the type strains were analyzed by DNA–DNA hybridization. These strains were cultured by shaking in tryptic soy broth (TSB; Difco Laboratories Inc., Detroit, MI, USA) for 16 h at 50°C, and the cells were collected by centrifugation. Genomic DNA was extracted from the cells and purified using the methods described by Hamamoto [17] and Kawamura [18]. DNA–DNA hybridization was performed as described by Ezaki et al [19] using a microplate reader (GENios, Tecan Trading AG, Männedorf, Switzerland). DNA extraction, purification, and hybridization were performed by TechnoSuruga Laboratory Co. Ltd., Shizuoka, Japan.

### Evaluation of bacterial tolerance to NH_4_Cl and NaCl

The tolerance of TAT105 and the *B. kokeshiiformis* and *B. thermolactis* type strains to NH_4_Cl and NaCl were evaluated by cultivation method using liquid and solid media. To evaluate their tolerance to NaCl, the bacteria were cultured in TSB and tryptic soy agar (TSA) containing 1% to 12% NaCl. Tolerance to NH_4_Cl was evaluated using yeast extract-ammonium (YA) medium, which was developed for cultivation of TAT105 [12]. YA medium contained 0.5% yeast extract (Difco Laboratories Inc., USA), 0.1% Na_2_HPO_4_, 0.03% KH_2_PO_4_, 0.1% CH_3_COONa, and 0.5 to 1.5 M NH_4_Cl. The agar concentrations in the solid media (YA agar) were varied within the range of 3% to 6% depending on the NH_4_Cl concentration. For liquid culture, each strain was inoculated into 7 mL of YA medium in a 50 mL test tube with a cotton plug. The tube was shaken in a slanted position at 55°C for 24 h. For solid culture, each strain was streaked onto YA agar and incubated at 60°C for 2 d.

### Estimation of TAT bacterial concentrations in the composts and PCR-RFLP

Composts prepared from typical materials (cattle manure, swine manure, poultry manure, bark, rapeseed oil cake, and excess sludge from the wastewater purification plant) were collected from various parts of Japan. The composts were obtained from different manufacturers and locations. The concentrations of TAT bacteria in these composts were determined by cultivation on YA1 agar. This agar consists of YA medium with 1 M NH_4_Cl and 3.5% agar and was developed for quantification of TAT bacteria in swine manure composts [13]. Each compost was suspended in sterilized physiological salt solution (PSS, 0.85% NaCl) and homogenized with a blender (AM-5; Nihonseiki Kaisha Ltd., Tokyo, Japan) at 18,000 rpm for 15 min. After serial dilution using PSS, the suspension was inoculated on YA1 agar. The inoculated plates were incubated at 60°C for 2 d, and grown colonies were counted. Separately, the dry matter content in the compost was measured by incubation at 105°C for 2 d, so that TAT bacterial concentration in the compost (CFU/g DM) could be calculated. In total, 93 composts were examined: 23 cattle manure, 18 swine manure, 21 poultry manure, 15 bark, 9 rapeseed oil cake, and 7 excess sludge.

Some strains of TAT bacteria were randomly picked up from the colonies on YA1 agar, purified, and subjected to PCR-RFLP as described in the previous study [13]. Each strain was cultured in YA medium containing 100 mM NH_4_Cl, and genomic DNA was extracted from the collected cells. 16S rDNA was amplified by PCR using a universal primer set [20]. Amplified DNA was digested with HaeIII or HinfI. Digests were subjected to electrophoresis, and PCR-RFLP profiles were determined.

## RESULTS

### Phylogenetic placement of TAT105

**Figure 1:**
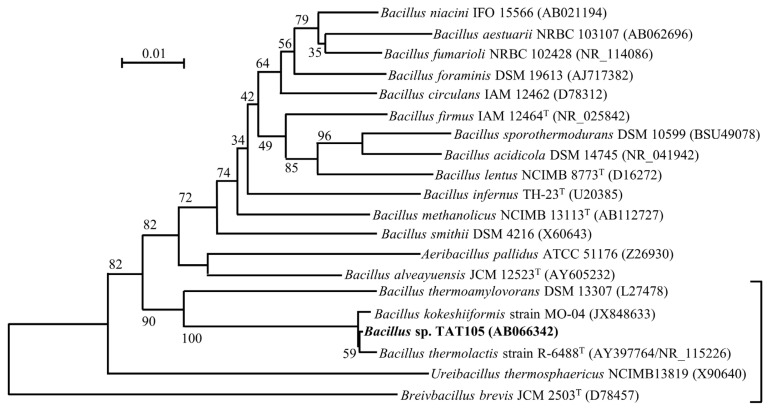
Phylogenetic tree comprising *Bacillus* sp. TAT105 and existing species in the genus *Bacillus* and related genera based on 16S rDNA sequence data. The scale bar indicates 0.01 substitutions per nucleotide position. Accession numbers of sequences in the International Nucleotide Sequence Database Collaboration (INSDC) are shown in parentheses. The region indicated by the bracket on the lower right corresponds to the tree shown in Figure 2.

The phylogenetic tree shown in Figure 1 includes TAT105 and its most closely related species. Among these species, the recently registered *Bacillus thermolactis* [21] and *Bacillus kokeshiiformis* [22] were closest to TAT105, displaying 99% similarity in their 16S rDNA sequences.

**Figure 2:**
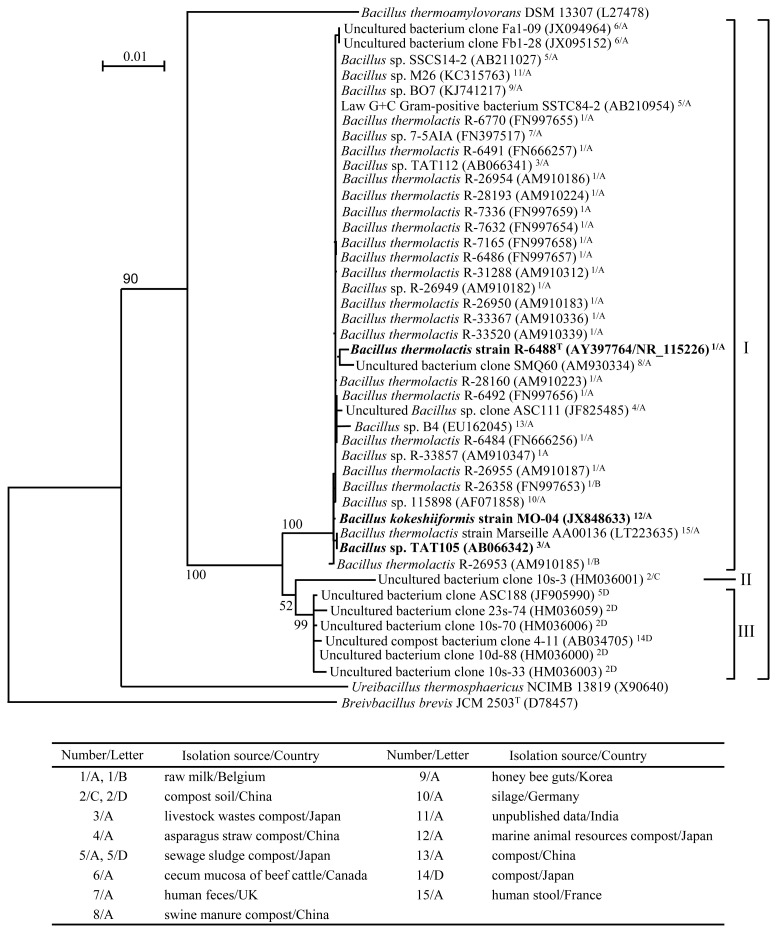
Phylogenetic tree comprising *Bacillus* sp. TAT105 and its closest relatives based on 16S rDNA sequence data. The scale bar indicates 0.01 substitution per nucleotide position. Accession numbers of the sequences in INSDC are shown in parentheses. The strains within the bracket on the right-side show 99% sequence identity with TAT105 16S rDNA. Superscript numbers and letters following the parentheses indicate the isolation sources and the countries where the strains and clones were isolated (1–15) and the types of restriction map of HaeIII and HinfI in 16S rDNA (A–D), respectively. The strains and clones with the same number were isolated by the same research group. The quick table below the phylogenetic tree indicates correspondence between the numbers/letters and isolation sources/countries. Roman numerals I–III indicate the two clusters (I and III) and a strain (II) distinguished with these types: I with A or B, II with C, and III with D. The frames to the left of I and III indicate the ranges of their respective clusters.

Variants of *B. thermolactis*, several *Bacilli*, unidentified bacteria, and uncultured clones close to TAT105 have been isolated worldwide (Figure 2). Some of these strains and clones, including TAT105 and *B. kokeshiiformis*, were isolated from the composts of various materials.

The restriction site analysis using NEBcutter V2.0 revealed that there were four types of specific restriction maps of HaeIII and HinfI in 16S rDNA (types A to D) among the strains shown in Figure 2. TAT105 and the *B. kokeshiiformis* and *B. thermolactis* type strains showed type A. Type B, observed in two variants of *B. therrmolactis*, was very similar to type A, but contained an additional HinfI restriction site quite close to the 3′-end of the 16S rDNA. Types A and B corresponded to the PCR-RFLP profile of HaeIII- or HinfI-digested 16S rDNA of the TAT bacteria detected in swine manure composts using YA1 agar in previous study [13].

Based on the branching of the tree and the types of restriction maps, these strains were classified into two clusters (I and III) and a strain (II): I with type A or B, II with type C, and III with type D. TAT105, *B. kokeshiiformis*, and *B. thermolactis*, and their variants were included in cluster I.

Table 1 shows the results of the DNA–DNA hybridization. The DNA–DNA relatedness was above 70% between TAT105 and *B. thermolactis* but below 70% between TAT105 and *B. kokeshiiformis*. The DNA–DNA hybridization is used for the identification of the strain with 16S rDNA showing higher than 98.7% sequence similarity with those of the existing species [23], and the DNA–DNA relatedness between the independent species should be lower than 70% [24]. *B. thermolactis* and *B. kokeshiiformis* were recognized as different species with low DNA–DNA relatedness (45%) [22]. Based on these results, TAT105 was considered to be a variant of *B. thermolactis*.

### Tolerance to NaCl and NH_4_Cl of TAT105 and its closest relatives

Table 2 shows the tolerance of TAT105 and the *B. thermolactis* and *B. kokeshiiformis* type strains to NaCl and NH_4_Cl. The tolerance of TAT105 to NaCl on TSA was slightly higher than those of two type strains, although the tolerances of these three strains to NaCl were similar in liquid culture in TSB. The tolerances of these strains to NH_4_Cl were similar in both the agar and liquid media. The colonies of two type strains on TSA and YA agar including YA1 agar were morphologically identical to that of TAT105 observed in the previous study [13].

### Distribution of TAT bacteria in various composts

**Figure 3:**
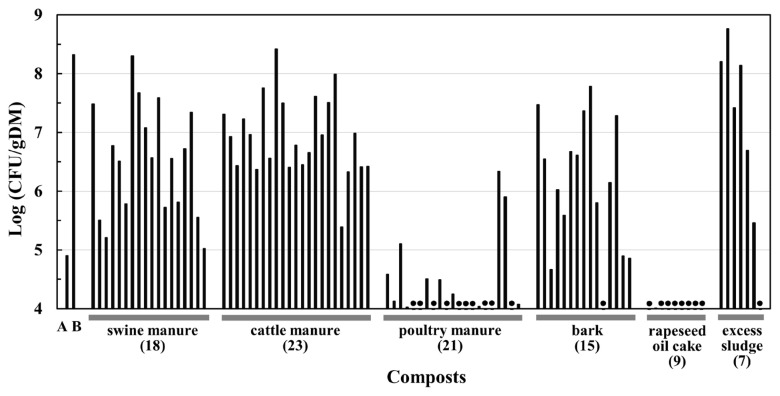
TAT bacterial concentrations in various composts. Columns A and B indicate the average TAT bacterial concentrations of the control treatment without the addition of TAT105 in swine manure composting tests [14]. A and B represent the concentrations at the start and end of composting, respectively. The remaining columns show the concentrations of TAT bacteria in the composts examined in this study. Filled circles indicate values less than 10^4^ CFU/g DM. The raw materials and number of the compost samples are shown on the horizontal axis.

Figure 3 shows TAT bacterial concentrations in the composts investigated in this study. Cattle manure, swine manure, bark, and excess sludge composts contained TAT bacteria at concentrations above 10^5^ CFU/g DM, with several exceptions. Some of them had concentrations (10^8^ to 10^9^ CFU/g DM) close to those of swine manure composts produced in the composting tests in a previous study [14]. In contrast, TAT bacterial concentrations in most poultry manure and rapeseed oil cake composts were below 10^5^ CFU/g DM.

**Figure 4:**
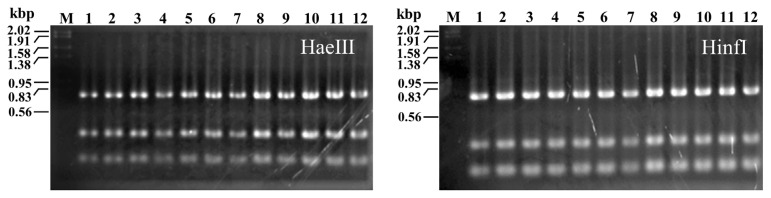
PCR-RFLP profiles of 16S rDNA of TAT105, *Bacillus thermolactis*, *Bacillus kokeshiiformis*, and TAT bacteria in the composts digested with HaeIII and HinfI. Lanes: M, marker (Marker 2, Nippon Gene Co Ltd., Tokyo); 1, TAT105; 2, *B. thermolactis* strain R-6488^T^; 3, *B. kokeshiiformis* strain MO-04; 4–12, TAT bacteria isolated from swine manure (4–6), cattle manure (7–9), and excess sludge (10–12) composts.

All TAT bacterial colonies that grew on YA1 agar were morphologically identical to those of TAT105 and the type strains of *B. thermolactis* and *B. kokeshiiformis*. Some colonies of TAT strains were randomly picked up and subjected to PCR-RFLP analysis. The results showed the PCR-RFLP profiles of HaeIII- or Hinf-digested 16S rDNA of these strains were identical to those of TAT105 and the *B. kokeshiformis* and *B. thermolactis* type strains (Figure 4). These profiles were matched with those presumed to be from type A or B of the 16S rDNA restriction map shown in Figure 2 and were different from the profiles presumed to be from types C and D.

## DISCUSSION

In this study, phylogenetic analyses of TAT105 were conducted, and the results of these analyses were used to verify the identity of TAT bacteria, previously observed in composting of swine manure [13,14]. Phylogenetic analysis based on 16S rDNA sequence revealed that TAT105 is most closely related to *B. thermolactis* and *B. kokeshiiformis*, and DNA–DNA hybridization results indicated that TAT105 is a variant of *B. thermolactis* (Figure 1; Table 1). Close relatives of TAT105, including *B. kokeshiformis* and *B. thermolactis*, are widely distributed in nature, and some of them have been isolated from various composts. These relatives showed specific HinfI and HaeIII restriction map in their 16S rDNA (type A or B), and were assigned to cluster I in Figure 2. The *B. thermolactis* and *B. kokeshiiformis* type strains showed similar tolerance to NaCl and NH_4_Cl as TAT105 (Table 2). Coorevits et al [21] reported that the *B. thermolactis* type strain had a low tolerance to NaCl (<0.1%) but showed a higher tolerance (≤6% in TSB and ≤10% on TSA) in this study.

The *B. thermolactis* and *B. kokeshiiformis* type strains and TAT bacteria in the composts examined in this study formed colonies on YA1 agar that were morphologically identical to that of TAT105. Additionally, these type strains and TAT bacteria in the examined composts, which were randomly picked up from the colonies on YA1 agar, showed PCR-RFLP profiles identical to those of TAT105 (Figure 4). These profiles correspond to type A or B of the restriction map in Figure 2. These results suggested that TAT bacteria detected using YA1 agar corresponded to the strains in cluster I shown in Figure 2, and the strains in this cluster had characteristics of TAT bacteria, as well as those of TAT105 and the *B. thermolactis* and *B. kokeshiiformis* type strains.

The TAT bacterial concentrations in the composts clearly differed according to the raw materials of composts (Figure 3). In most cattle manure, swine manure, bark, and excess sludge composts, TAT bacteria were detected at 10^5^ to 10^9^ CFU/g DM. In contrast, the concentrations in most poultry manure and rapeseed oil cake composts remained at concentration of <10^5^ CFU/g DM. The high TAT bacterial concentration in the compost was the result of their increase at the high temperatures during composting, as observed in the composting of swine manure in previous studies [13,14]. This suggests that the adaptability of TAT bacteria to composting depends on raw material of the compost, and these bacteria are generally adaptable to composting of the four compost materials mentioned above.

This characteristic bacterial group may include some strains that have useful functions, other than TAT105. Considering the presumed adaptability of TAT bacteria to composting of these four materials, this bacterial group seems a promising object for screening of the strain applicable to composting of these materials for desirable control of composting.

The direct addition of cultured microbes to a specific environment is an application of microbial biotechnology. One example of this is the use of biological additives for the treatments of animal wastes. Many biological additives are available and are advertised to have some effect in the treatments; however, little information has been provided on the microorganisms in these additives and their functions in the treatments [25–27]. The search for microorganisms that are useful for waste treatment and their application has been conducted, but few studies have validated their adaptability to these treatments [9,10,28].

This study verified the general identity of the TAT bacteria. Their distribution in the assessed composts suggested that they are highly adaptable to composting of several materials, including cattle and swine manure. Additionally, the high selectivity of YA1 agar for TAT bacteria and its usefulness as a simple detection method for TAT bacteria using YA1 agar was reconfirmed, as in previous study [13]. The results described here, and the method used can be utilized in subsequent studies on this bacterial group. The screening of useful TAT bacterial strains and their application to composting of TAT-adaptable materials, including evaluation of their adaptability and applicability, should be conducted in the future.

## Figures and Tables

**Table 1:** DNA–DNA relatedness between TAT105 and *Bacillus thermolactis* or *Bacillus kokeshiiformis*

Immobilized DNA	Labeled DNA^1)^		

	1	2	3
1	100	72 (3.8)	65 (3.8)
2	84 (7.2)	100	-
3	56 (2.0)	-	100

1) Strains: 1, TAT105; 2, *B. thermolactis* R-6488^T^; 3, *B. kokeshiiformis* strain MO-04.

The average percentages obtained from experiments performed three times are shown.

The values in parentheses represent the SD.

**Table 2:** Tolerance of *Bacillus* sp. TAT105, *Bacillus thermolactis*, and *Bacillus kokeshiiformis* to NaCl and NH_4_Cl

Item	Strains^1)^		

	1	2	3
NaCl (%)			
In TSB	≤5	≤6	≤6
On TSA	≤11	≤10	≤8
NH_4_Cl (M)			
In YA medium^2)^	≤0.5	≤0.5	≤0.5
On YA agar^2)^	≤1.4	≤1.3	≤1.3

TSB, tryptic soy broth; TSA, tryptic soy agar.

1) 1, TAT105; 2, *B. thermolactis* R-6488^T^; 3, *B. kokeshiiformis* strain MO-04.

2) YA medium, yeast extract-ammonium medium developed for cultivation of TAT105 [12]; YA agar, a solid medium consisting of YA medium and 3% to 6% agar.
